# Resilience in Sports: Sport Type, Gender, Age and Sport Level Differences

**DOI:** 10.3390/ijerph18158196

**Published:** 2021-08-03

**Authors:** Cecilia Blanco-García, Jorge Acebes-Sánchez, Gabriel Rodriguez-Romo, Daniel Mon-López

**Affiliations:** 1 Faculty of Physical Activity and Sport Sciences (INEF), Universidad Politécnica de Madrid, 28040 Madrid, Spain; cecilia.blanco@upm.es (C.B.-G.); daniel.mon@upm.es (D.M.-L.); 2 Faculty of Health Sciences, Universidad Francisco de Vitoria (UFV), 28223 Madrid, Spain; j.acebes.prof@ufv.es; 3 Centro de Investigación Biomédica en Red sobre Fragilidad y Envejecimiento Saludable (CIBERFES), 28029 Madrid, Spain

**Keywords:** resilience, sport, physical activity, gender, age, BRS

## Abstract

There seems to be a broad consensus that there is a positive correlation between resilience and sport performance. However, different studies show divergent results on the role played by certain variables in this relationship. This study aimed to analyze the possible relationships between resilience levels and the practiced sport according to gender, age, and competitive level of the athletes in 1047 competitive athletes from five different sports (handball, basketball, volleyball, athletics, and judo). Resilience was assessed with the Spanish version of the Brief Resilience Scale (BRS). Results of independent samples t-tests or analysis of variance (ANOVA) showed no significant differences on the level of resilience according to the practiced sport or the competitive level. However, the analyses of covariance (ANCOVA) showed that they were related to the gender and age of the athletes, being higher in males than in females, and there was a positive correlation with age. These results seem to suggest the convenience of using differentiated strategies, according to gender and age, when working on all those protective factors that could allow the athlete to perform better when facing adversity in the competitive environment.

## 1. Introduction

Psychological resilience has been conceptualized as both a trait and a process. From a trait perspective, resilience is defined as a set of fixed and stable characteristics that enable individuals to adapt to various significant sources of stress or trauma [1,2]. These characteristics or individual differences are commonly referred to as protective factors. In contrast, resilience is also seen as a dynamic response, which enables positive adaptation in the face of exposure to adversity [3]. In this case, the influence of personal characteristics will vary according to the situation and the moment. Thus, the response to stressors will essentially be a process developed in the context of person–environment interaction [4,5]. Moreover, the reaction to stressful events may also vary across the life cycle. It will depend on the timing of the individual, the intentionality of the risk factors, and the availability of protective factors [6]. Thus, despite its different conceptualization as a trait and a process, resilience can be considered a psychological characteristic that promotes positive adaptation to adverse processes or periods.

In the physical activity and sports context, aspects such as effort, struggle, sacrifice, overcoming challenges, rivalry, evaluation, risk of injury, assimilation of defeat, and, in short, facing and overcoming numerous adverse and stressful situations are, to a greater or lesser extent, are inherent to the practice. These environmental demands generate significant stress processes with which athletes have to cope [7]. For this reason, psychological resilience has been identified as a relevant variable in this context, arousing a growing interest as an object of research over the last decades [7,8,9,10,11,12]. However, several authors point out that, unlike what happens in other fields of study such as clinical or health care, where individuals are “forced” to show resilience qualities to maintain their functioning in the face of potentially traumatic events, athletes, on the other hand, choose to face challenging situations voluntarily [7,13]. Therefore, due to the contextual specificity of resilience [3], the findings of studies carried out in other fields are not always extrapolated to the context of physical activity and sport. Research is needed in this specific context [13].

In the physical activity and sports field, the study of psychological resilience has been approached from a dual perspective. On the one hand, several studies aimed to analyze the possible relationships between the practice of physical activity and sport and the development of resilience, considering that this practice can provide individuals with a series of experiences and positive personal qualities that enable its improvement [14,15,16]. In this line, several studies with university students associate the practice of physical activity (moderate physical activity or that performed in sufficient quantity to obtain health benefits especially) with higher levels of resilience [17,18]. In the health context, Ho et al. [19], in a study with adolescents, concluded that physical activity is positively related to mental health, with resilience being the only significant mediator in this relationship. In the sport-specific domain, White and Bennie [16] concluded that gymnasts and coaches perceived that participation in gymnastics developed resilience, life skills, self-efficacy, and self-esteem. These findings support the idea that youth sport may be a suitable avenue for resilience development [20], as noted by Martinek and Hellison [14] in their review of physical activity and sport-based prevention programs with at-risk youth.

Furthermore, different researchers and professionals have been interested in the role of psychological resilience in competitive sport and its relationship with sports performance [13,21,22,23,24]. They seek to identify, among other aspects, factors that may predict resilience or how athletes may become more resilient. In this respect, there seems to be a broad consensus that there is a positive relationship between high levels of resilience and optimal sports performance. To cite some examples, Hosseini and Besharat [22], with a sample of athletes from different sport modalities, found that resilience was positively associated with sport achievement and psychological wellbeing and negatively associated with psychological distress. Similarly, Yang et al. [24], in their study with high school taekwondo players, concluded that resilience improved the ability of these athletes to use psychological skills, having positive effects on performance enhancement.

Different theoretical models have been developed to explore and explain the relationship between psychological resilience and optimal sports performance [13,25]. Of these, the grounded theory of psychological resilience and optimal sport performance [13] is probably the most influential for understanding the resilience process in high-performance athletes [9]. Fletcher and Sarkar [13] point out that exposure to highly stressful stimuli is an essential feature of the stress–resilience–performance relationship in competitive sport. Athletes tend to perceive stressors as opportunities for growth. They evaluate stressful situations as a motivating challenge and not as a threat. These stress factors can be related to sport performance (e.g., injury, rivalry, pressure), to the sport organization in which athletes operate, and to personal events outside sport (e.g., family or work problems). Athletes’ positive appraisal of adversity depends on several psychological factors that, in good balance, protect them from the possible negative effects of stressors, leading to optimal sports performance. These protective factors are positive personality, motivation, confidence, concentration, and perceived social support [7,12,13].

Although there is consensus on the positive correlation between psychological resilience and optimal sports performance, the same is not true when it comes to determining whether resilience is equally relevant in different sport disciplines, or whether there are sports or sport modalities that promote greater development of resilience. Consequently, this leads to differences in the resilience levels of their respective practitioners. The results of the studies that have been carried out in this regard show divergent results. Chacón-Cuberos et al. [26] analyzed the levels of resilience in athletes of three sports modalities (football, handball, and skiing), and no statistically significant differences were found according to the practiced modality. Boghrabadi et al. [27] compared resilience levels between high-level athletes playing team or individual sports and nonathlete university students. Although athletes showed higher mean values of resilience than nonathletes, they found no statistically significant differences among these groups. Bingol and Bayansalduz [28] also found no association between resilience levels and sport modality in team sports (volleyball, basketball, handball, and football) and individual sports (boxing, wrestling, Muay Thai, and taekwondo). In contrast, the study conducted by Reche-García et al. [29] concluded that practitioners of combat sports had significantly higher levels of resilience than the individual or team sports athletes.

Likewise, there is no clear consensus on the role played by gender and age concerning resilience levels in the sport context. Regarding age, Codonhato et al. [30], in their study with athletes who participated in the Paraná Open Games 2012, found higher levels of resilience among older athletes. However, other studies also conducted in the field of sports [26,29,31] did not find associations between resilience levels and age. In terms of gender, the results are also discrepant. Zurita-Ortega et al. [32] and Biricik and Sivrikaya [33] found significantly higher resilience values in males than in females when studying a sample of judo athletes and a sample of university students from the Faculty of Sport Sciences, respectively. In the same vein, Patsiaouras [34], comparing resilience levels in a sample of volleyball players by gender, found significant differences in favor of men on variables such as self-efficacy, solution seeking, and goal orientation. In contrast, Reche-García et al. [29] only found significant differences in the levels of resilience according to gender among team sports practitioners, being higher in the case of women. On the other hand, other studies in the context of sport have found no association between the two variables [22,27,28].

The discrepant results regarding the possible differences in the levels of resilience of athletes according to gender, age, or the type of practiced sport, highlight, as pointed out by various authors, the need to develop more studies aimed at identifying the relationships between these variables, as well as their possible dependence on a competitive level or experience in competition [9,11,30,35]. Likewise, the convenience of investigating these aspects in large samples of athletes has also been pointed out [9,29,30,35,36] because many of the studies carried out to date have been conducted on relatively small samples, making comparisons between groups difficult, with possible generalization of their results.

Considering all of the above, this study aimed to analyze the possible relationships between the levels of resilience of a large sample of competitive athletes and the sport practiced, as well as to explore their dependence on gender, age, and competitive level of the athletes. The hypotheses were as follows: (1) combat sports athletes (judo) would have higher levels of resilience than those of individual sports (athletics) or team sports (basketball, handball, and volleyball); (2) levels of resilience would be significantly higher among athletes with a higher competitive level; (3) male competitors would have higher levels of resilience than females; (4) there is a significant positive correlation between age and the level of resilience.

## 2. Materials and Methods

### 2.1. Design and Participants

We conducted a descriptive and cross-sectional study. The study population were athletes, active competitors over 18 years of age, from five different sports: basketball, handball, volleyball, athletics, and judo. The sample was selected in a nonprobabilistic and incidental way. The snowball sampling technique was used, based on the researchers’ personal contacts and social networks. A total of 1645 surveys were collected. Only the questionnaires of the athletes who met the following inclusion criteria were analyzed: (1) athletes must have an active federative license and have officially competed during 2020; (2) athletes must be Spanish and live in Spain during the survey. In addition, incomplete surveys, athletes under 18 or over 39 years old, and technical staff were excluded from the study. The final sample consisted of 1047 athletes divided between five sports: basketball (*n* = 165), athletics (*n* = 242), handball (*n* = 165), volleyball (*n* = 158), and judo (*n* = 317). Additionally, athletes were classified into three categories of sports: team sports (*n* = 488), individual sports (*n* = 242), and combat sports (*n* = 317), and into two types of sports: team sports (*n* = 488) and non-team sports (*n* = 559).

Men (*n* = 589, 56.3%) and women (*n* = 458, 43.7%) were 24.37 ± 6.01 and 24.05 ± 5.78 years old, respectively. These percentages fit almost perfectly with the real distribution of the Spanish federated population in our five sports analyzed (men 57.6% and women 42.4%) [37]. Regarding the sport level, 167 athletes were selected by their national teams in the last two years, while 880 athletes were not. All participants signed an informed consent form before completing the survey. All surveys were anonymous to verify the sincerity of the participants’ responses. The sample distribution by gender and sport, sports category, and sport type and the resilience values are showed in Table 1.

### 2.2. Instrument and Variables

Variables were distributed in two areas: demographic and psychological variables. Demographic questions were based on previously used questionnaires [38], and their variables were age (years), gender (male or female), residence (Spain or other countries), nationality (Spanish or other nationality), sport level (selected by the national team in the last two years (yes or no)), sport (basketball, athletics, handball, volleyball, and judo), sports category (team sport, individual sport, and combat sport), sport type (team sport and non-team sport), and sport relationship (athlete or technical staff).

On the other hand, the psychological variable measured was resilience, using the Brief Resilience Scale (BRS) [39] Spanish validated version [40]. This survey is composed of six Likert scale-type questions ranging from 1 (totally disagree) to 5 (totally agree). The BRS reliability in our study was α = 0.74, a value that can be considered acceptable.

### 2.3. Procedure

The final version of the survey was formatted into a Google Forms questionnaire and was uploaded and shared on the Google online survey platform. A link to the electronic survey was distributed to personal contacts via WhatsApp and email. Additionally, the link was published on other social media platforms (Facebook, Twitter, and official federation web pages) using the snowball sampling technique [41].

The survey included an introductory page describing the background, the aims of the study, and the ethics information. The questionnaire was available online for a maximum of three months during the 2020 year. The questionnaire was open and anonymous to verify the sincerity of the answers. Participants had an unlimited amount of time to complete the survey. Once the deadline for admitting surveys was closed, these were reviewed to remove contradictory responses (checking the congruence between the data provided by the players), empty questionnaires, or duplicated surveys (checking two or more submissions with the same responses in a short period of time), deleting one response from the database.

### 2.4. Statistical Analysis

The data were described by arithmetic mean (*M*) and standard deviation (*SD*). The normal distribution of the variables was checked using the Kolmogorov–Smirnov tests. Linear regression was performed to determine the age influence on the BRS values. Independent sample t-test was used to compare groups of sport level, gender, and sport type (team and non-team sports). The analyses of the differences by sport and sport category (team, individual, and combat sports) were carried out using an analysis of variance (ANOVA). The post hoc comparison between groups were made using the Games–Howell Test. When statistical differences were found, the effect size was calculated using Hedges’ g values and establishing three cut-off points: low effect (0.20), medium effect (0.50), and large effect (0.80), or with the partial eta squared (*η*^2^; 0.01 = small, 0.06 = medium, 0.13 = large) [42]. The confidence interval for the effect size was set at 95%. Additionally, to analyze resilience differences between genders, sport, sport type, sport category, and sport level groups, controlling the age’s effect, an analysis of the covariance (ANCOVA) test was used. IBM SPSS Statistics software (SPSS 25.0. IBM Corp., Armonk, NY, USA) was used for the mathematical calculations. The level of significance was set at *p* < 0.05.

## 3. Results

Age was found as a significant predictor of the resilience values *F*_(1,1045)_ = 15.32; *p* < 0.001; *β* = 0.12. Accordingly, age was included in all the comparisons. The analysis by pair groups (gender, sport level, and sport type) showed the following differences. Men had higher resilience scores than women (*p* < 0.001; Hedges’ g = 0.405), but there were no differences in the age (*p* > 0.05). No differences were found between sport levels in resilience nor in age (*p* > 0.05). In addition, team sports were younger than non-team sports (*p* = 0.019; Hedges’ g = 0.144), but both sport groups had the same resilience scores (*p* > 0.05) (see Table 2).

The analysis by sport category showed differences in age (*F*_(2,1044)_ = 13.78; *p* < 0.001), but not in the resilience values (*p* > 0.05). The post hoc analysis showed differences between combat sports and individual sports (*p* < 0.001) and team sports (*p* < 0.001). Additionally, when the analysis was carried out by sport, differences were found in age *(F*_(4,1042)_ = 7.91; *p* < 0.001), but not in the resilience values (*p* > 0.05). The post hoc analysis showed differences between judo and basketball (*p* = 0.005), athletics (*p* < 0.001), and handball (*p* < 0.001), but not with volleyball (*p* > 0.05) (see Table 3).

Finally, an ANCOVA test was made to check differences between groups when the age was controlled, confirming differences in resilience scores between genders (*F*_(1,1044)_ = 41.98; *p* < 0.001; *η*^2^ = 0.039), but no differences between sports (basketball, athletics, handball, volleyball, and judo), sport categories (team sports, individual sports, and combat sports), sport types (team and non-team sports), and sport level (all *p* > 0.05).

## 4. Discussion

Our results show that the levels of resilience do not present significant differences according to the practiced sport, nor on the competitive level (selected for the national team in the last two years vs. not selected). However, resilience seems to be related to the gender and age of the athletes, being higher in males than in females, and having a positive correlation with age.

Regarding the possible relationships between resilience levels and the practiced sport, the study recently conducted by Reche-García et al. [29] found significant differences in the resilience levels of 278 athletes (194 men and 84 women) who practiced individual, team, or combat sports, being significantly higher in the case of combat sports. No differences were found between the individual and team sports. Additionally, as noted by Piskorska et al. [43], in most sports disciplines, during competition, maximum effort is required from athletes in situations of mental stress and great physical fatigue. However, in the case of combat sports, other circumstances of a more specific nature, usually present during competition, must also be added, which means that the psychological and personality traits of the athletes, as well as their cognitive abilities, are particularly important aspects of performance in combat sports. Some of these characteristics are the physical contact and direct attack on the opponent’s body, the usual precompetition weight loss, and the risk of experiencing pain and possible injuries, as well as the persistent fear of failure in the face of the opponent. Therefore, considering all these aspects and the conclusions of the study by Reche-García et al. [29], the first hypothesis in this research was that combat sports (judo) practitioners would present higher levels of resilience than those of individual sports (athletics) or collective sports (basketball, handball, and volleyball).

However, our results do not allow us to confirm this hypothesis. No significant differences in the levels of resilience according to the practiced sport, neither when considering each of the five sports analyzed individually (basketball, handball, volleyball, athletics, and judo), nor when grouping them by sport category (individual sport, team sport, and combat sport) or type of sport (team sport vs. non-team sport). Moreover, these results were confirmed controlling the possible effect of age (a significant predictor of resilience levels) and when not doing so, despite age significant differences between some of the groups. In all the sports analyzed, mean resilience values were very similar and were included, in all cases, in the “normal” resilience range (3.00–4.30), according to the cut-off points for the interpretation of BRS scores [39,44]. Therefore, the results obtained in the present study would be in line with those of previous studies that also found no association between levels of resilience and the practiced sport [27,28,45]. Indeed, these studies did not explicitly compare resilience levels in combat sports versus individual or team sports, as per Reche-García et al.’s study [29]. However, in the study conducted by Bingol and Bayansalduz [28] on 777 athletes (313 women and 464 men), with a minimum experience of five years of practice, there were no significant differences between individual sports; all of them were combat sports (boxing, wrestling, Muay Thai, and taekwondo) and team sports (volleyball, basketball, handball, and football).

The second hypothesis was that resilience levels would be significantly higher among athletes with a higher competitive level. However, when comparing the resilience levels of athletes who had been selected for the national team during the last two years with the rest of the competitors, no significant differences were found. Therefore, this second hypothesis is not confirmed either. There is a broad consensus in the literature that resilience and sports performance are positively related [13,21,22,23,24,25]. It might seem that our results are contrary to this consensus. However, optimal sports performance does not necessarily imply being among athletes with the highest competitive level (high-level athletes). Rather, it refers to the athlete, regardless of his or her category or level, being able to effectively and efficiently use those capabilities and resources at his or her disposal to achieve the best possible sporting results. Therefore, we believe that our findings do not oppose those positive associations between resilience and sports performance that, in general, have been described in previous studies, but rather that they are complementary. In other words, the positive relationships between resilience and sports performance could occur regardless of the competitive level of the athletes. In addition, our results coincide with one of two found studies in which possible differences in the levels of resilience were specifically analyzed according to the competitive level of the athletes [34,46]. Thus, as in the present study, Castro-Sánchez et al. [46] did not find statistically significant relationships between both variables in a sample of 43 athletes (football, handball, and winter sports), classified by their competitive level into professionals, semiprofessionals, amateurs, and hobbyists. In contrast, Patsiaouras [34], with a sample of 98 volleyball players, found that players with a higher competitive level were able to concentrate more on finding solutions and overcoming problems, compared to lower level players.

Regarding the possible associations between gender, age, and resilience, the results obtained in the present study do seem to confirm the two hypotheses put forward, i.e., that male athletes would have higher levels of resilience than females and that the older the athletes, the higher the levels of resilience. Concerning age, our results coincide with those of the study by Codonhato et al. [30], with 150 athletes (107 in team sports and 43 in individual sports) who participated in the 2012 Paraná Open Games. These authors found higher levels of resilience among older athletes. Likewise, several studies with a general population sample also confirm a positive association between age and resilience [40]. As noted by Codonhato et al. [30], the relationship of age with resilience may be an outcome consistent with the concept of resilience itself. If the development of resilience is a process that occurs over time, depending on lived experiences, it is to be expected that older individuals have faced a greater number of adversities in different life contexts, which could increase their levels of resilience. However, contrary to the results obtained in the present study, other previous studies with specific samples of athletes [26,29,31] did not find associations between resilience levels and age.

Concerning gender, our results are in line with those of other studies [32,33,34] that also found significantly higher levels of resilience in male than in female athletes. In particular, Zurita-Ortega et al. [32], with a sample of 148 Chilean judoists, concluded that, in men, increased physical self-concept led to higher levels of resilience than in women. Biricik and Sivrikaya [33] studied the resilience levels of 278 university students from the Faculty of Sport Sciences, finding significantly higher values in males than in females. Patsiaouras [34], in his study with volleyball players, also found higher levels of resilience in men than in women. Women showed less determination than men in overcoming obstacles that interfered with the achievement of their volleyball goals. In contrast, men found it easier to move away from problematic thoughts and develop solution-finding thinking skills. Furthermore, the relationships found in our study between gender and resilience, despite having a small effect size (g = 0.405 and *η*^2^ = 0.039), were further confirmed when controlling for the effect of age. In any case, other studies have also found no association between the gender of athletes and their levels of resilience [22,27,28].

It is possible that this lack of clarity about the relationships between gender, age, and resilience may be due, as Lee et al. [2] point out in their meta-analysis, to the relatively small and homogeneous samples used in different studies; as well as in the sports domain, it also analyzed such relationships in other contexts. Consequently, it might seem that our results only add to this controversy without providing greater clarity, but we believe that this is not the case. The sample of the present study was neither small nor homogeneous. Following suggestions from other previous studies on resilience in sport [9,29,30,35,36], a large sample of competing athletes was analyzed to try to overcome these limitations. Therefore, it is possible that our results on the relationships between resilience, gender, and age in the sport context point in a direction that should be confirmed in future research.

However, the present study is not without other limitations. The first of these is related to the instrument used to assess resilience. The BRS scale [39] is a psychometric test not developed for the sport context, and based on a self-report questionnaire. In this regard, several authors have criticized the fact that resilience in sport is measured based on general scales, developed in other fields, considering that the factors assessed are specific to the context in which they arise and therefore cannot be easily generalized to other populations [8,12,47]. They, therefore, point to the need to create sport-specific auto-report scales that capture the breadth and depth of resilience in this context. However, such a specific scale has not yet been developed [47], so researchers continue to commonly use general self-report measures such as the Connor–Davidson Resilience Scale (CD-RISC; [1]) or the BRS itself [39]. Despite these limitations, the BRS was chosen, as it was intended to obtain information from several competing athletes. As it is a short scale (six items), it was considered convenient to minimize response time, participant fatigue, and, consequently, the loss of information and quality of responses. Furthermore, the BRS seems to be a valid and reliable scale to assess resilience in the sports domain [48], although it has not been developed specifically for sport. For this reason, it should be noted that the use of this instrument may not be capturing the nuances of sport-related performance resilience [7]. Future research should take into account the theoretical and conceptual framework specific to resilience in the sport context [7,8]. On the other hand, another limitation of the present study is related to its cross-sectional and descriptive design, which does not allow us to establish causal relationships between the analyzed variables. Likewise, no information was obtained on the race of the participants, so the sample studied may lack diversity in this representation. Future studies should take this aspect into account and consider the possible comparison of the results with a nonathlete population.

Despite these limitations, we believe that our study provides greater clarity than that existing to date on the possible associations of resilience with the type of practiced sport, the competitive level, gender, and age of the athletes. In this line, we consider that its main strength lies in the breadth of the sample studied and its heterogeneity, with a significant number of participants in each of the categories of the different variables analyzed and a wide age range, ranging from 18 to 39 years old. To date, there are very few quantitative studies on resilience in the field of sport with samples with similar characteristics to the one used in this study [9].

Likewise, we believe that the results of this study also provide relevant information, from a practical point of view, for coaches, psychologists, physical trainers, sports medical practitioners, physiotherapists, and other support staff working in the field of competitive sport. The relationships found between resilience, gender, and age of the athletes seem to suggest the convenience of using differentiated strategies, depending on these variables, when working on all those protective factors (individual, social, and environmental) that could allow the athlete to cope with stressors and, consequently, perform better in the face of adversity in the competitive environment.

## 5. Conclusions

In the sample of competing athletes who participated in this study, resilience is not associated with the practiced sport (basketball, handball, volleyball, athletics, and judo). Nor do associations appear when grouping these sports by sport categories (individual sport (athletics), team sport (basketball, handball, and volleyball) and combat sport (judo)) or types of sport (team sport (basketball, handball, and volleyball) vs. non-team sport (athletics and judo)). In addition, resilience is not related to the competitive level of the athletes (selected for the national team in the last two years vs. not selected). However, gender and age of the athletes are related to the levels of resilience, being higher in men than in women and increasing with age.

## Figures and Tables

**Table 1:** Age and resilience descriptive data by gender, sport, sports category, and sport type.

Sports/Sports Category/Sports Type	Gender	BRS		Age	
		*M*	*SD*	*M*	*SD*
Basketball (*n* = 165)	men (*n* = 118)	3.49	0.66	23.81	5.49
	women (*n* = 47)	3.19	0.76	23.28	4.69
Athletics (*n* = 242)	men (*n* = 139)	3.51	0.66	23.69	5.27
	women (*n* = 103)	3.23	0.80	22.77	5.16
Handball (*n* = 165)	men (*n* = 104)	3.51	0.59	23.32	5.00
	women (*n* = 61)	3.36	0.72	23.03	4.45
Volleyball (*n* = 158)	men (*n* = 52)	3.51	0.64	23.92	5.46
	women (*n* = 106)	3.23	0.71	24.77	5.97
Judo (*n* = 317)	men (*n* = 176)	3.59	0.68	26.03	7.21
	women (*n* = 141)	3.24	0.70	25.16	6.60
Team sport (*n* = 488)	men (*n* = 274)	3.50	0.63	23.65	5.29
	women (*n* = 214)	3.26	0.72	23.95	5.34
Individual sport (*n* = 242)	men (*n* = 139)	3.51	0.66	23.69	5.27
	women (*n* = 103)	3.23	0.80	22.77	5.16
Combat sport (*n* = 317)	men (*n* = 176)	3.59	0.68	26.03	7.21
	women (*n* = 141)	3.24	0.70	25.16	6.60
Team sport (*n* = 488)	men (*n* = 274)	3.50	0.63	23.65	5.29
	women (*n* = 214)	3.26	0.72	23.95	5.34
Non-team sport (*n* = 559)	men (*n* = 315)	3.55	0.67	25.00	6.52
	women (*n* = 214)	3.26	0.72	23.95	5.34
Total (*n* = 1047)	men (*n* = 589)	3.53	0.65	24.37	6.01
	women (*n* = 458)	3.25	0.73	24.05	5.78

Notes: *M* = mean; *SD* = standard deviation; *n* = number of athletes; age = age in years; BRS = resilience scores.

**Table 2:** Age and resilience comparisons by groups of gender, sport level, and sport type.

Variable	Comparison Groups				*p*	Effect Size	Confidence Interval 95%	
	*M*	*SD*	*M*	*SD*		Hedges’ g	*LL*	*UP*
	Men		Women					
BRS	3.53	0.65	3.25	0.73	<0.001	0.405	0.282	0.529
Age	24.37	6.01	24.05	5.78	0.394			
	Low sport level		High sport level					
BRS	3.41	0.70	3.40	0.73	0.940			
Age	24.09	5.88	24.99	6.01	0.071			
	Team sport		Non-Team sport					
BRS	3.39	0.68	3.42	0.72	0.611			
Age	23.78	5.31	24.63	6.36	0.019	0.144	0.022	0.265

Notes: *M* = mean; *SD* = standard deviation; *n* = number of athletes; *p* = level of significance; Hedges’ g = effect size; *LL* = lower limit; *UP* = upper limit; age = age in years; BRS = resilience scores.

**Table 3:** Age and resilience comparisons by sport and sport category.

Sports Category/Sports	Age		*p*	Effect Size	Confidence Interval 95%		BRS	
	*M*	*SD*		Hedges’ g	*LL*	*UP*	*M*	*SD*
Team sport	23.78 ^A^	5.31	<0.001	0.31	0.167	0.452	3.39	0.68
Individual sport	23.30 ^A^	5.23	<0.001	0.374	0.205	0.542	3.39	0.74
Combat sport	25.64	6.95					3.43	0.71
Total	24.23	5.91					3.40	0.70
Basketball	23.66 ^B^	5.27	0.005	0.308	0.119	0.497	3.40	0.70
Athletics	23.30 ^B^	5.23	<0.001	0.374	0.205	0.542	3.39	0.74
Handball	23.21 ^B^	4.79	<0.001	0.386	0.196	0.576	3.45	0.64
Volleyball	24.49	5.80					3.32	0.70
Judo	25.64	6.95					3.43	0.71
Total	24.23	5.91					3.40	0.70

Notes: *M* = mean; *SD* = standard deviation; *n* = number of athletes; *p* = level of significance; Hedges’ g = effect size; *LL* = lower limit; *UP* = upper limit; age = age in years; BRS = resilience scores. ^A^ = significant differences with combat sport; ^B^ = significant differences with judo.

## Data Availability

The data are available for those who want to see it with justified reasons. Kindly contact the corresponding author.
